# Physical intelligence as a new paradigm

**Published:** 2021-04-26

**Authors:** Metin Sitti

**Affiliations:** Physical Intelligence Department, Max Planck Institute for Intelligent Systems, 70569 Stuttgart, Germany; Institute for Biomedical Engineering, ETH Zurich, 8092 Zurich, Switzerland; Koç University, School of Medicine & College of Engineering, 34450 Istanbul, Turkey

**Keywords:** Physical Intelligence, mechanics, meta materials, multistability, mechanical memory, mechanical computation

## Abstract

Intelligence of physical agents, such as human-made (e.g., robots, autonomous cars) and biological (e.g., animals, plants) ones, is not only enabled by their computational intelligence (CI) in their brain, but also by their physical intelligence (PI) encoded in their body. Therefore, it is essential to advance the PI of human-made agents as much as possible, in addition to their CI, to operate them in unstructured and complex real-world environments like the biological agents. This article gives a perspective on what PI paradigm is, when PI can be more significant and dominant in physical and biological agents at different length scales and how bioinspired and abstract PI methods can be created in agent bodies. PI paradigm aims to synergize and merge many research fields, such as mechanics, materials science, robotics, mechanical design, fluidics, active matter, biology, self-assembly and collective systems, to enable advanced PI capabilities in human-made agent bodies, comparable to the ones observed in biological organisms. Such capabilities would progress the future robots and other machines beyond what can be realized using the current frameworks.

## 1 Introduction

Robots and autonomous cars have been entering more and more to our daily lives, such as homes, on and inside our body, buildings, roads, factories, hospitals, agricultural fields, mines and nuclear plants. These machines are aimed to perform various tasks in such unstructured and complex real-world environments. They need to be intelligent to achieve such goals autonomously, safely and robustly. Here, ‘intelligence’ can be functionally defined as their ability to perceive (sense, interpret), control (decide, plan, predict, regulate), act (move, change, affect, coordinate) and learn (adapt, evolve, acquire experience, infer) continuously and automatically. Artificial intelligence (AI) field emerged to create intelligence for physical and virtual agents by introducing brain-inspired [1] and abstract computational intelligence (CI) [2] methods. However, intelligence of physical agents, such as human-made machines and biological organisms, is not only enabled by their CI in their brain, but also by their physical intelligence (PI) encoded in their body. Therefore, it is essential to advance the PI of physical agents as much as possible, in addition to their CI. Here, PI can be defined as *physically encoding sensing, actuation, control, memory, logic, computation, adaptation, learning and decision-making into the body of an agent.* We do not call it body intelligence since some agent bodies can also have embedded CI in specific cases, such as an octopus with a mini-brain in its each arm in addition to its central brain [3]. On the other hand, PI can also exist among teams and swarms of agents through coupled physical interactions to create collective, self-organized and other multi-agent intelligent behavior.

**Fig. 1:**
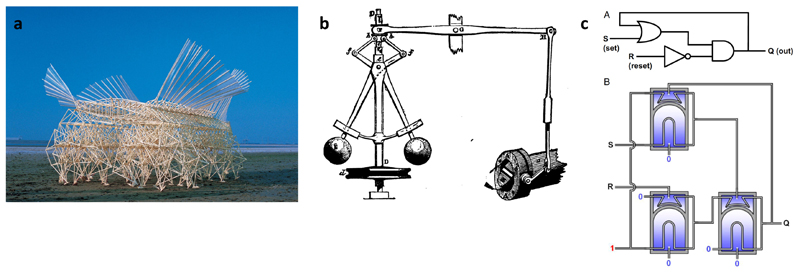
Physical intelligence (PI) components and enablers on a physical agent body interacting with its environment. Such body PI is also coupled to the computational intelligence (CI) in the brain, where the embodied intelligence (EI) field investigates the tight coupling between the body and brain.

PI differs from the embodied intelligence (EI) field, which has been around for a long time [4–8]. While PI only focuses on the physical intelligence encoded in the body, EI focuses on the tight coupling between an agent’s body and brain (and the environment). For example, EI-based evolutionary body variation approaches aim to couple the body morphology and function with CI, tasks and environment in multiple levels [9]. On the other hand, PI is related to EI in the cases where PI is complementing or simplifying CI, especially in CI-dominated agents at large length scales. Therefore, in such agents, both fields complement each other to understand how body can create intelligence physically itself while interacting with the environment (PI) and how such body is tightly coupled with brain (EI) (see Fig. 1).

Some PI methods have been already investigated and partially realized in various fields, such as mechanics (e.g., multistable structures, metamaterials, origami, kirigami), materials science (e.g., smart, stimuli-responsive and functional materials), robotics (e.g., soft robots, small-scale robots), mechanical design, fluidics, active matter, biology, self-assembly and collective systems. However, such progress has only introduced limited and simple PI capabilities so far, while there is an urgent need for more advanced PI capabilities for enabling intelligent machines operating autonomously in real-world conditions. Therefore, PI, as a new paradigm, aims to synergize above and other relevant fields and merge them under the same umbrella to enable advanced PI capabilities in human-made machines, comparable to the PI observed in biological systems. Thus, enhanced PI capabilities would progress the future robots and other machines beyond what can be realized using the current dominantly CI-based and limited PI-based frameworks. A recent commentary introduced a PI concept and proposed a methodology of educating the researchers to gain skills for the creation of PI [10]. Differently, this perspective article aims to define in detail when PI can be more significant and dominant in agents at different length scales and how bioinspired and abstract PI methods can be created in agent bodies.

Animals have evolved to create neural intelligence (NI), which is the biological counterpart of CI, and PI to survive and operate autonomously in unstructured and resource-limited real-world environments. As one of the approaches to introduce advanced PI methods, we can get inspired by the PI methods utilized in biological organisms. Moreover, understanding the PI and NI methods and their interactions in biological organisms could inspire us in designing PI and CI methods for human-made physical agents operating in similar real-world environments and length scales with similar functions.

## 2 Brief history of physical intelligence and neural intelligence during the evolution of biological systems

Life on earth started with diverse unicellular organisms in the ocean 1-3.5 billion years ago. Intelligent behavior of such single cells came from their biological materials, structures, sensing, signal processing, communication, memory, adaptation and actuation capabilities with a very limited complexity. Thus, intelligence in biological systems started with PI first. Then, when multicellular larger organisms started to appear around 0.5 billion years ago, evolution started to differentiate various specialized cell types to create more complexity and more advanced intelligent behavior. As one of the types of specialized cells, neurons evolved to transmit the electrical and chemical signals from all sensory cells in the organism’s body to a central nervous system (spinal cord and brain), create cellular networks to process and store information in such system, and transmit motor signals from such system to control all motor cells in the body, e.g., contract the muscle cells and synthesize hormones and other vital substances.

The first decentralized neural nets (a number of neurons spread apart the body that allow the organism to respond to physical contact) appeared in *Cnidaria,* comb jellies *(Ctenophora)* and jellyfishes around 500 million years ago. Later, such simple and decentralized neural nets evolved into complex and centralized neural networks in the brain and spinal cord of vertebrates, invertebrates and other animals at different length scales ~0.25–300 million years ago to create NI, in addition to their more enhanced PI. In the special case of unicellular organisms, all plants, and some invertebrates and multicellular organisms with no neurons (e.g., fungi, sea sponges, slime molds, placozoa), only PI enabled the intelligent behavior.

NI level of animals is determined by many different factors in their brain, such as their brain size, mass, cortical neuron number, neuron packing density, intraneuronal distance, memory capacity and duration, and axonal conduction velocity. Human beings appeared around 250 thousand years ago with a 1.4 kg brain, which consists of a complex network of total ~86 billion neurons with the highest number of cortical neurons (~16 billion) in nature and consumes much more energy than other animal brains [11]. NI in the human brain and spinal cord has enabled the most complex and abstract capabilities, such as creating and using tools, causal inference, linguistics, planning, decision making, abstract and creative thinking, and imagination. At the same time, the human body has much more advanced PI capabilities than other animals to significantly enhance the human intelligence further. Our advanced individual and collective NI have taken us to the top of the food chain in nature by enabling unprecedented linguistic, social, cultural, scientific, technological, and artistic progress. Overall, using their NI and PI, the biological systems at different length scales have evolved to operate and survive just “good enough” in their complex, diverse and resource-limited natural environments.

## 3 Physical intelligence versus computational intelligence in agents at different length scales

For autonomous human-made agents, while many AI and machine learning researchers are working intensely to create CI tools and methods as good as or even better than the biological NI, above brief overview can show us that advanced PI capabilities have also been indispensable to have in agents operating in unstructured complex environments in different length scales, like in the biological systems.

**Fig. 2:**
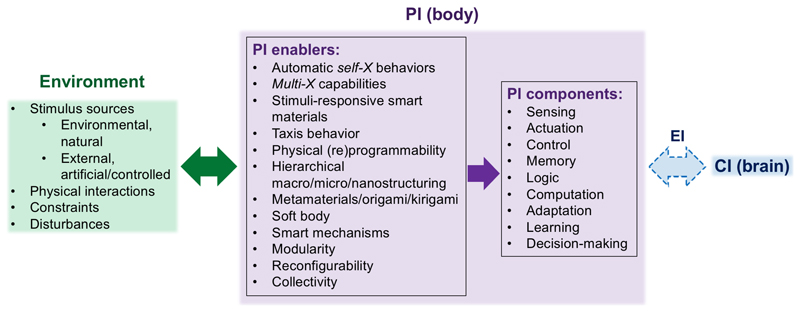
Autonomous human-made machine examples using mainly physical intelligence. (a) Theo Jansen’s human-size kinetic art machines called strandbeest [12] (Animaris Currens Ventosa 1993, photo: Adriaan Kok) using self-powering/actuation using wind energy harvesting-based motion generation, mechanical sensing and self-regulation for avoiding the sea water, and walking multi-legged mechanisms and feet optimized for a robust and efficient walking on sandy beaches. (b) Self-regulating mechanical control system example from the history: centrifugal governors were used as self-regulating mechanical control systems in James Watt’s steam engines from 1788 (Adapted from [13]. Copyright, George Routledge and Sons). Similar self-regulating mechanical control systems have been implemented these days in some vehicle transmissions and record players. (c) A soft pneumatic logic circuit and memory, outputting a binary value of pressure depending on the most recent non-zero input signal of pressure (Aapted from [14]. Copyright 2019, National Academy of Sciences).

At large (centimeter or meter) length scales, most of the current human-made agents have advanced computational capabilities with the current scientific and technological advancements to use the available CI tools dominantly to operate in unstructured environments. In such CI-dominant agents, increasing the PI in the agent body can minimize the load of, simplify or complement CI. Especially, at very complex (e.g., fully soft-bodied), purely mechanical (no electronics) or ultra-high-speed machines, PI can significantly enhance or even fully replace the CI at the large scales. For example, the kinetic sculpture artist Theo Jansen’s purely mechanical, legged machines at the human-size scale (Fig. 2a) [12] harvest the wind energy to walk on sandy beaches while they can sense and avoid the sea water using mechanical sensing, computation and control mechanisms. He calls such artistically created machines as new artificial forms of life and they are great examples of autonomous human-scale machines purely using PI to operate in unstructured environments.

At smaller (millimeter) length scales with limited on-board computation and powering capabilities at the current technological stage, PI becomes as important as CI. If you like to design and build an insect-scale flying robot with flapping or rotary wings, in addition to a fast and low-power microprocessor (brain) with many minimalist and fast CI tools, you need to integrate PI as much as possible on the robot’s body by specialized optical flow [15] and gyroscopic sensors with many specialized filters, signal processors and self-regulators embedded in the hardware, impact-resistant robust exoskeletons against high-impact crashes with the environment [16], multifunctional and adaptive body morphology [17] and structures, and air drag-reducing body shapes.

At sub-millimeter length scales down to micron (single cell-size) scale, current autonomous machines do not have on-board computation and powering capabilities so that PI becomes the only or main option [18]. Therefore, for example, autonomous microswimmers need to interact with their operation environment to get their fuel or harvest energy from their environment, and sense and follow specific stimuli in their microenvironment to reach to a target location and deliver their cargos, such as drugs, using their stimuli-responsive and shape-programmable smart materials and structures.

In all of these cases, typically, PI is more specialized and relatively simple while CI is more general purpose and complex. Moreover, in very special harsh environments, such as space, nuclear power plants and everywhere after a nuclear reactor or war disaster, where extreme thermal, mechanical and radiation conditions can hinder the operation of electronic devices, PI and mechanical devices would be the only option for physical agents at all length scales.

## 4 Methods to create physical intelligence in physical agents

To achieve advanced PI capabilities in physical agents at different length scales for given specific applications and tasks, there are many possible enablers and design considerations. In general, there are some key enablers that can be implemented to create advanced PI capabilities in a physical agent (Fig. 1):

- Encoding automatic *self-X* capabilities (e.g., self-adaptation, self-response, self-regulation, self-propulsion, self-healing, self-powering, self-cleaning, self-degrading, self-growing, self-replicating, self-cooling, self-oscillation, self-assembly, self-organization) in the agent body with no or minimal CI interference by integrating passive or active smart materials, structures or mechanisms.
- Encoding *multi-X* capabilities (e.g., multifunction, multistability, multilocomotion, multiterrain, multimodality, multiphysics) in the agent body, where the same or different material compositions, structures and mechanisms and physical forces or effects can induce multiple functions or behaviors at the same time to minimize the sensing, actuation, control and learning complexity for each specific function and behavior. Designing such *multi-X* capabilities in agent bodies require evolutionary algorithms-type computational nonlinear optimization methods since achieving such capabilities simultaneously would induce many design parameter conflicts.
- Encoding various other advanced physical capabilities and properties in the agent body, such as mechanical logic operations, memory, computation and decision making, re-configurability, modularity, physical (re)programmability, smart structuring (e.g., multistable structures, metamaterials, origami, kirigami, tensegrity), hierarchical multi-length scale structuring, smart mechanisms, taxis behavior, and collective and emergent behavior. Such capabilities would require minimal or no CI.

For above PI approaches, possible functional smart materials for physical agent bodies consist of stimuli-responsive or multifunctional synthetic polymers (e.g., hydrogels, liquid crystal elastomers, shape memory polymers, soft composites, magneto-elastic materials, piezoelectric polymers), metals (e.g., shape memory alloys, liquid metals), ceramics (e.g., piezoelectric ceramics), textile fabric, biomaterials (e.g., chitosan, cellulose, gelatin, silk, proteins, cells, tissues), and micro/nanomaterials-based composites (e.g., carbon fiber composites, ferrofluids, magneto/ electrorheological fluids, micro/nanoscale particle/disc/wire/fiber/ tube/crystal-filled polymers).

Below includes more detailed and specific example approaches to create PI for physical agents through designing the body material compositions, structures and mechanisms.

### 4.1 Passive materials-based body compositions, structures and mechanisms

Passive isotropic or anisotropic materials and structures in the agent body, which do not respond to external stimuli, can be designed to enable self-adaptive, self-regulatory, self-degrading, self-cleaning, and other autonomous behaviors. For example, precisely tuned leg, foot and body joint compliance and damping can enable very fast, self-regulating and stable dynamic running locomotion and surface transitions in a six-legged robot on a rough and 3D complex terrain autonomously without any real-time feedback control of each leg, like cockroaches [19]. As another example, designing a flapping-wing based flying robot where there is a precisely tuned passive torsional spring on the wing base [20] could enable passive rotation of the wing without requiring additional actuation and control at very high speeds and frequencies. Although such underactuated design enables only a specific wing rotation timing (e.g., delayed rotation), such minimalist design enables reduced control complexity and significant weight reduction, which makes CI easier and enable easier miniaturization down to the real insect size scale.

**Fig. 3:**
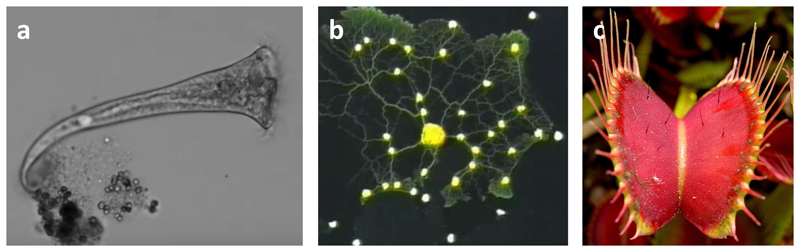
Inspired by biological microhair structures, diverse multifunctionalities can be encoded to the agent body surfaces using synthetic microfiber arrays: surface adhesion and friction of vertical or angled microfiber structures can be controlled using active or passive, vertical or lateral mechanical load control; microfiber arrays can be designed to super-repel or super-attract specific or all liquids in the operation environment; fibers can reduce the fluid or air flow-induced drag forces on the body; they can control the heat and electrical conductance of the agent surface with or without contacting to another surface; they can be used as a flow or contact sensor if a transduction material is integrated to the fiber base or structure; and they can induce ciliary motion-based fluid or solid object transport if they are made of an active material.

Like passive biological micro-hairs on the animal and plant bodies, passive microfiber arrays with different materials, diameters, aspect ratios, spacings, orientations and tip ending shapes covering the body surfaces can enable multiple simultaneous functions for physical agent bodies (Fig. 3). First, such microfiber surfaces can be used for reversible, fast, energy-efficient, compact and mechanically controlled adhesion and friction on a wide range of surfaces or objects in the environment, similar to the biological foot-hairs of geckos, spiders and many flying insects. Such surfaces can enable PI for surface crawling and climbing robots and robotic soft grippers by mechanically controlled (even switchable) adhesion and friction [21,22]. Next, such microfiber surfaces can be designed to be super-repellent or super-attractant against water, oil and other liquids in the environment, as a liquid-wetting control method [23]. Liquid super-repellent fiber surfaces enable self-cleaning of the agent body surface, where any solid dirt on the fiber surfaces can be cleaned autonomously by rain or other water droplets in the environment [24]. Also, such liquid super-repellent property is advantageous to adhere to wet surfaces and objects by pushing out the liquid out of the fiber–surface interface and prevent any surface ice formation when operating in freezingly cold environments. Moreover, such fiber surfaces can simultaneously reduce aerodynamic drag on the robot body (saving locomotion energy) by passively controlling the air flow and circulation on the body surface [25], reduce hydrodynamic drag under water (until a limited depth due to their given finite hydrostatic pressure resistance) by trapping air bubbles between fibers. As another functionality, such microfibers can be also used for air or liquid flow sensing and control [26], if a sensory transduction material is integrated to the base of or on the hairs. Next, they can be used to control body surface temperature by preserving the heat on the surface (like the hairs on our head) and electrical conductivity with any other contacted surface. Finally, if active polymeric materials are used as the hair material, the micro-hairs can be used as active microcilia, which could pump fluids and transport solid objects and mucus-like very viscous liquids directionally [27–29].

Passive body materials and morphology can be designed in specific ways also to enable energy-efficient, sustainable, robust, easy-to-control, safe and self-adaptive behaviors. High strength-to-weight-ratio materials (e.g., carbon fiber composites, abalone shell-inspired nacreous micro/nanostructured composite materials) can reduce the inertial force-related energy consumption of agents without sacrificing their mechanical strength much. Using energy-dissipating materials, structures or mechanisms on a flying, perching or other machine outer body can make it self-adaptive and safe against high mechanical impacts with the environment by absorbing shocks passively [30]. Next, using specific biocompatible and non-immunogenic synthetic materials, such as hydrogels and zwitterionic polymers [31], and patient’s own red blood cell [32] or other extracted biological materials, medical robots can be safe and stealthy against the body immune cells for long durations inside the human body. If the agent body material is biologically (e.g., enzyme-based), optically (e.g., sunlight), temperature-based, or chemically (e.g., pH) degradable in a given specific operation environment, such physical agents can be designed to degrade and dissolve inside their operation environment (e.g., inside the human body, in indoors/outdoors, in space) in a pre-programmed duration after its function is over [33–35]. Thus, we could have self-degrading, environment-friendly and safe agents, like the biological organisms. Finally, if the agent body is designed to be neutrally buoyant under water, that would make the swimming control easier in three dimensions (3D) since the depth of the agent can be kept constant passively with no extra control or CI. Since typical synthetic robot materials are much denser than water, the bare material selection is difficult to create neutral buoyancy, where a 3D body design with an air or other gas reservoir is required, similar to some gas-filled fish bladders for buoyancy control [36].

As another PI design approach, utilizing *metamaterials* (artificial materials with unique properties that do not exist in nature and their properties are due to their solid periodic structure and not material composition) in the agent body. Metamaterials can have mechanical, photonic, electromagnetic, acoustic and other multiple functions. Periodically structured mechanical metamaterials can behave as auxetic materials with a negative Poisson’s ratio such that they contract transversely rather than expand when compressed longitudinally. Also, they can behave like a fluid although they are solid structures, and manipulate electromagnetic waves by blocking, absorbing or bending them. For example, mechanical metamaterials with a multistable architecture can trap elastic strain energy for significantly enhancing energy absorption [37]. The mechanism of energy absorption stems solely from the structural geometry of the bistable beam elements, and is therefore both material- and loading rate-independent.

Origami, kirigami and tensegrity structures can be also used to enable PI properties in the agent body. Origami can turn a 2D sheet of any material with programmed folding lines into complex 3D shapes. Self-folding origami structures and mechanisms can produce shape-programmable robotic bodies [38], exoskeletons [39], wings [40] and joints [41]. Next, kirigami is similar to origami by replacing the fold lines with cut lines in 2D sheets, which can also create 2D-to −2D and 2D-to −3D shape-morphing agent body structures and mechanisms. As an example, kirigami robotic skins can simplify the required actuation for a soft crawling robot [42], and liquid crystal networks-based [43] and elastomer-based kirigami structures can be stimulated by light and heat change to create complex 2D and 3D shapes. Finally, tensegrity structures, which maintains a stable volume in space through the use of discontinuous compressive elements (struts) connected to a continuous network of tensile elements (cables), can enable extremely lightweight yet strong mechanical structures for agent bodies [44,45].

Finally, by designing functionally graded (e.g., stiffness/ modulus gradient) or anisotropic (e.g., directional stiffness) physical properties on the agent body passive material and structure, the agent can have higher performance, survival and locomotion. For example, a soft jumping robot’s functionally graded soft robot body can have a stiffness gradient, spanning three orders of magnitude in modulus, from a rigid core to a soft exterior [46]. Such stiffness gradient can enable a reliable interfacing between rigid and soft body components to reduce any failure at the soft-rigid component regions through stress reduction at the interface of materials mismatched in compliance. Also, it can improve the robot’s ability to survive after-jumping landings by increasing its body energy/shock absorbance and reduced peak stresses. As another example, a directionally stiff flapping robot wing design can be very stiff in the flapping bending direction at its leading edge and very compliant in the torsional rotation direction at its trailing edge so that it could have a passive wing rotation at the trailing edge tuned for enhanced aerodynamic lift generation without another wing rotation mechanism and actuator [47].

### 4.2 Active and stimuli-responsive smart materials-based body compositions, structures and mechanisms

For enabling PI, active and stimuli-responsive materials that have a programmed response to remotely controlled or environmental stimuli can enable autonomous sensory, actuation, powering, self-healing and other PI functions on the agent body. Here, remotely controlled stimuli could be laser light and electrical, acoustic and magnetic fields or gradients. Environmental stimuli can be mechanical stress (e.g., contact, air or fluid flow), changes in temperature, humidity and pH, sunlight, chemical and biological (e.g., enzymes, glucose) compounds, and specific chemical triggers. Active body material’s response to such stimuli could be change in its shape, volume, stiffness, damping, density, electrical and heat conductivity, color, transparency, wettability, adhesion, and friction.

Stimuli-responsive body materials can enable self-sensing of the operation environment (environment sensing) or the location, movement, and action of parts of the body itself (proprioception) since a specific environmental stimulus or body part motion can induce a specific programmed response on the material. Through such response the body can indirectly sense the environment, which could be communicated with CI system or create an autonomous self-response. For example, bonding of specific biochemical molecules (e.g., pathogens) in the environment to specifically designed molecules covered on a flexible microcantilever beam surface at the microscale can induce surface strain and beam deflection, which can be detected optically, piezoresistively, magnetically or piezoelectrically [48]. Also, controlled or external mechanical stress on a micro/nanostructured hydrogel robot skin can be used to self-regulate its structural color to camouflage the robot in a given environment [49] like octopi and chameleons and self-sense the external stress optically. Next, both biological and synthetic agents can be programmed to have specific *taxis* behavior to sense the local stimuli in their operation environment and autonomously respond to them using their stimuli-responsive materials, structures or mechanisms (e.g., change their motility to go towards or escape from such stimulus source). Such taxis behavior can be due to chemical, light, oxygen, pH, stiffness, moisture, fluid flow, pressure and temperature gradients in the local environment, the Earth’s magnetic field (25 to 65 μT depending on the specific location) or externally applied magnetic field, electrical field and gravity, which are called chemotaxis, phototaxis, aerotaxis, pH-taxis, durotaxis, hydrotaxis, rheotaxis, barotaxis, thermotaxis, magnetotaxis, galvanotaxis and gravitaxis, respectively. Using such taxis behavior, for example, bacteria-driven microswimmers can autonomously aggregate inside the tumor cells since the anaerobic bacteria attached to their robot body can sense and be attracted towards the hypoxic, oxygen-deprived (aerotaxis) and low pH (pH-taxis) microenvironment of tumor cells.

In addition to the typical on-board actuators, active smart stimuli-responsive materials (e.g., magnetic, piezoelectric, shape memory, and other stimuli-responsive polymer materials) can be used for actuating the agent body using environmental natural stimuli (e.g., sun light, air/liquid flows, pH, temperature, humidity, ionic and chemical changes) or externally-controlled human-made stimuli (e.g., magnetic fields, acoustic waves, mechanical vibrations, flows and a focused laser beam) for different PI purposes. First, the agent can move in an efficient and agile way for locomotion in its complex environment, manipulating its environment, reconfiguring itself, adapting, and realizing other specific motor actions with minimal energy consumption autonomously or by external stimuli control. For example, using external magnetic fields, programmed magneto-elastic soft composite actuators can enable shape-programmable and self-adaptive robot body dynamics for multimodal locomotion [50] and multifunctional [51,52] behavior and safe direct physical interaction with human beings inside or outside their body. As an example of environmental stimuli-driven body actuation, when a clothing on the body of a person has an increased temperature due to a high metabolic activity, shape memory polymer material-based fabric structures can self-respond and self-fold (bend upwards autonomously) to enable more air access/flow in the heated regions and cool down the person fast [53]. Moreover, using responsive materials, specific functions of the agent can be triggered autonomously. For example, by environmental or externally controlled stimuli, the body material can change its volume or molecular composition and release loaded internal drugs or other cargoes autonomously for on-demand cargo delivery in a target location [54–57].

Moreover, active materials can be used to actively tune the stiffness and damping dynamics for adaptive and high-performance robot locomotion, manipulation and environment interaction for new tasks or dynamically changing environmental conditions [58,59]. For example, while soft body materials enable complex shape deformations with minimal control inputs and designs, they cannot exert too much force to the environment, which could be problematic for a specific task requiring also high force output. Then, active stiffness or damping tuning on a soft body can enable adaptive large shape deformations in its compliant/soft state or high force output at the rigid/hard state at a given time. Such stiffness- or damping-tunable materials can be phase changing materials, such as liquid metals (changing liquid–solid phase change as a function of low temperature difference) [60], shape memory polymers [61] and magneto/electrorheological fluids [62].

Next, the body can have an autonomous self-propulsion using the stimuli in or interactions with the environment. Such self-propulsion can happen by chemical- and photocatalytic interactions of the smart robot body materials in fluidic media due to specific fuels/enzymes or light in the environment. Thermal gradients in the environment or local surfactant gradients induced by chemical reactions of the body smart material with the fluidic medium can respectively induce thermocapillary [63,64] and Marangoni effects [65,66] on the robot body to propel it autonomously. Moreover, micromotors research in the last decade has shown that self-generated gradients or fields, such as self-electrophoresis, self-diffusiophoresis, self-acoustophoresis, and self-thermophoresis, can be used for autonomous propulsion of micron-scale robots in liquid media [18]. For example, a bimetallic (gold–platinum) microrod can propel inside a hydrogen peroxide liquid medium since the catalytic interaction of these metals with the liquid medium induces asymmetric ion distribution on the rod body inducing its electrophoretic self-propulsion. Here, a chemical fuel, such as hydrogen peroxide, is needed, which can be also replaced by biological fuels or light. Especially, light-driven microswimmers have become possible by photocatalytic interactions of the swimmer body (e.g., a Janus particle half covered with gold or platinum and the other half being titanium oxide or carbon nitride) with light inducing self-electrophoresis or self-diffusiophoresis in water and biological fluids [67,68]. Such light-driven swimming microrobots also have self-sensing phototaxis property, where they can be steered or attracted towards a light source autonomously while being propelled by the same light source at the same time.

Self-powering smart materials embedded in the agent body can enable long autonomous operation durations and more sustainable and environment-friendly systems. Self-powering can be in two different ways. First, energy can be harvested from the environmental stimuli and forces on the agent, such as ambient light, external mechanical forces (e.g., fluid/air flows, mechanical vibrations), temperature gradients, humidity gradients, and tri-boelectrification [69–71] Such harvested power is typically very small, i.e., in the range of nW-mW, in a few centimeter and smaller length scale energy-harvesting devices, and they depend on the availability of the external stimuli/forces, which is random and time-dependent. Therefore, they are typically used to charge an existing on-board rechargeable power source, such as battery. Second, power can be self-generated by the chemical and physical interactions with the operation environment. For example, a robot operating in acidic environments, such as inside stomach [72] and the urinary tract [73], can have a potato battery-like power source to generate electrical power due to the acidic liquid medium. Also, fuel cells can generate power by using a continuous source of fuel and oxygen from air, and biofuel cells with specific enzymes can even generate power through the oxidation of blood sugars for robots operating inside the vascular system [74].

Self-healing (self-repairing) is another interesting PI capability inspired by biological systems, where we can design and embed stimuli-responsive material compositions and architectures to autonomously repair physical damages to the agent body to continue functioning similarly even after possible mechanical damages during interacting with its complex environment [75]. For example, microencapsulated healing agents can be embedded in the body material containing a catalyst capable of polymerizing the healing agent when a mechanical damage, such as crack, occurs on the body [76]. Also, polymeric body materials that contain dynamic covalent bonds could be triggered by environmental or external stimuli (e.g., UV light, temperature increase) after a mechanical damage to repair it promptly. In addition, natural or biosynthetic protein-based soft robot materials can self-heal against mechanical damages due to reforming their reversible physical bonds in a few seconds by the applied local temperature [77,78].

Anisotropic physical properties of the stimuli-responsive body materials, structures and mechanisms can induce programmable shape and other physical changes and sensory, actuation and logic behaviors when exposed to single or multiple stimuli. For example, cellulose structures can be 3D-printed by creating stiffness gradients to enable complex programmed deformations by environmental stimuli, such as humidity [79,80] and temperature [81] changes, which could be beneficial for intelligent robotic and architectural structures in outdoors. As another example, using liquid crystal elastomer materials on the body, their stiffness [82], optical property and stimuli-responsive contraction can be programmed in various directions precisely in 3D by aligning the director fields within the liquid crystal in 3D-programmed directions [83] so that any mechanical, optical, electrical or thermal stimulus can deform them in complex programmable shapes automatically [84].

### 4.3 Encoding memory into the agent body

Memory is an essential system component to enable CI [85] and self-adaptive PI behavior. In addition to electronic memory in typical CI systems, body can also have some limited physical memory capability for PI. First, temporary/permanent plastic deformation (e.g., memory foams, viscoelastic soft elastomers), granular jamming, or shape memory properties of the body material can enable volatile or non-volatile memorization of the mechanical deformations. Here, soft materials with internal granular jamming can keep a 3D deformation memorized for short or long durations for possible physical learning or adaptation. For example, a robotic soft gripper using suction-controlled granular jamming method can decide whether the next object to grasp has the same morphology by comparing it with the previously memorized jammed object morphology on the soft gripper. Moreover, shape memory alloys- and polymers-based bodies can be trained for specific shapes, and they can restore such specific memorized shapes at any of their deformed state after heating.

Non-volatile mechanical memory is also possible using bistable or multistable mechanical structures or metamaterials. A bistable mechanical beam can be used to have one-bit memory, ‘1’ (buckle up) and ‘0’ (buckle down) states, which can be switched optically [86] or using other magnetic, electrical or acoustic fields [87]. Also, two-bit mechanical memory is possible using more complex volumetric origami cells [88]. These origami units can work in a modular way, and they can interact with each other to demonstrate hierarchical, two-bit memory operations by exploiting the tunability of origami cells.

As another memory method embedded on the body, we can get inspired by the short-term chemical memory of PI-dominated unicellular bacteria during their chemotaxis, e.g., searching for food. Because of the temporal mode of chemical gradient sensing in bacterial chemotaxis, bacteria evolved to have a chemical short-term memory to adapt autonomously to time-varying stimuli to retain high sensitivity for chemical attractants over a wide range of (nanomolar to millimolar) concentrations [89]. Methylation of chemical receptors provides a sort of chemical memory, which allows the cell to compare the current ligand concentration to the past. Memory duration (e.g., 1–10 s) is a function of the stimulus strength and optimized by evolution to perform optimal comparisons of stimuli while swimming in the gradient. Methylation occurs at much slower time scales than other reactions involved in the system, thereby providing a memory mechanism, which allows the bacterium to remember its recent past state and compare its present situation to the past. Inspired by such different time-scale chemical/physical reactions, new short-term memory capability during chemotaxis and other stimuli-responsive taxis and behavior can be encoded in the agent body for enabling short-term memory-based self-adaptation capability.

### 4.4 Encoding control, logic and computation into the agent body

Physical control can be encoded into the agent body by using physical control systems, which could be mechanical, fluidic or pneumatic. After the industrial revolution, the steam engines used centrifugal governors to enable mechanical feedback and constant speed control. Such governors (Fig. 2b) used two balls swinging out and rising as the shaft rotational speed increased due to centrifugal forces, which decreased the valve opening (and thus decreased the fuel input to the engine and the shaft rotation speed) via a directly connected mechanical linkage until a predefined constant speed was achieved. Such mechanical feedback control system served as a linear proportional controller. Next, embedded small-scale fluidic or pneumatic devices can be used to control physical agents with no electronics. For example, in a soft-bodied octopus-inspired untethered robot, check valves, hydrogen peroxide fuel and catalytic reaction reservoirs, oscillator, reaction chambers, actuators and vent orifices were used as the mechanical replacements of electrical diodes, supply capacitors, electrical oscillator, amplifiers, capacitors and pull-down resistors, respectively, to control the robot fluidically [90]. Moreover, fluidic devices using microbubbles or microdroplets [91, 92] or soft pneumatic devices [14] can be also used to create combinational and sequential digital logic circuits and gates (Fig. 2c) for physical computation. Information can be physically represented by flow of electrons or ions, where it can also be manipulated through algorithmic manipulation of matter. DNA-based tile nanostructures can enable such algorithmic manipulation using self-assembly processes at the nanoscale [93] while ferrofluidic droplets can be used at the micronscale for physical information manipulation and computation [92].

In addition to above encoding of control and computation to the physical agent body, environmental stimuli can be used to directly and automatically control the action of physical agents as a PI-based control method. In this regard, if a self-sensing taxis behavior (e.g., phototaxis, chemotaxis, magnetotaxis, pH-taxis, thermotaxis) can be encoded to the agent body using smart material compositions, patterns and structures or integrated live biological cells (e.g., sensory neural cells, bacteria, algae), the agent can be guided towards to the stimulus source automatically in a stochastic manner. For example, bacteria-driven biohybrid microswimmers can use bacteria aerotaxis, chemotaxis, pH-taxis and also magnetotaxis (due to the magnetic nanoparticle loading inside the swimmer body) to control their swimming direction and target position [94,95]. Especially, the aerotaxis property of bacteria-driven microswimmers can automatically gather them in large numbers in the hypoxic (low oxygen) regions of the tumor cells [96] so that they could kill the cancer cells through the released loaded chemotherapeutic drugs from the swimmer body and also the recruited immune cells of the human body. On the other hand, if algae (plant cells) are used instead of bacteria for propelling the swimmers they could also have phototaxis property, which could accumulate them automatically in a target location using an ambient or illuminated light source [97]. Moreover, smart materials with a chemomechanical feedback control are possible by coupling chemical reactions and elastic reconfiguration, patterning chemical dynamics with mechanical stress, diffusion-coupled surface buckling, and hybrid polymer network-nanostructure systems on the physical agent body [98]. In the presence of multiple environmental stimuli, self-actuated agent body structures with geometries near bifurcation points associated with a transition between bistability and monostability can be also used to create bifurcation-based PI logic systems [99]. Here, anisotropically swelling polymeric structures made of multiple stimuli-responsive materials can create AND, OR, NAND and other type of logic operations and can be also used to program the self-actuation time by varying structural parameters.

### 4.5 Encoding adaptation, learning and decision-making into the agent body

Adaptation to changing environments, tasks and system parameters using passive/active, multifunctional and anisotropic materials, structures and mechanisms is one of the most significant PI methods to encode in the physical agents. Such physical self-adaptions could be through shape change, programming or reconfiguration, physical property (e.g., stiffness, heat transfer, electrical conductivity) change or tuning, and color adaptation for camouflage. For example, if a robot needs to move in a changing environment, which has also holes or cavities much smaller than its body size, a size- and shape-programmable soft-bodied or a shape-reconfigurable robot design could enable it to squeeze through such smaller cavities [100]. In another scenario, a robot could be needed to navigate inside a diameter-changing tubing system or the human vascular system with various vessel diameters, self-adaptation of the robot size or shape is critical for robust locomotion using surface crawling or rolling in such changing tubular environments. Moreover, legged robots walking or climbing on changing surface topographies and roughness can take advantage of a passively or actively tuning foot compliance, friction and shape adaptation to such changing surface conditions [101,102]. On the other hand, active tails of animals and robots can enable high stability and maneuverability during running, climbing, jumping and flying locomotion adapting to changing dynamic body forces/torques [103,104]. Finally, a soft robotic gripper can self-adapt to varying and unknown 3D object topographies due to its effectively compliant surface and grip them safely by its possible compliance increase through granular jamming [105] and other stiffness-tuning mechanisms or controlled surface adhesion and friction using dry fibrillar [106], suction or other attachment methods.

**Fig. 4:**
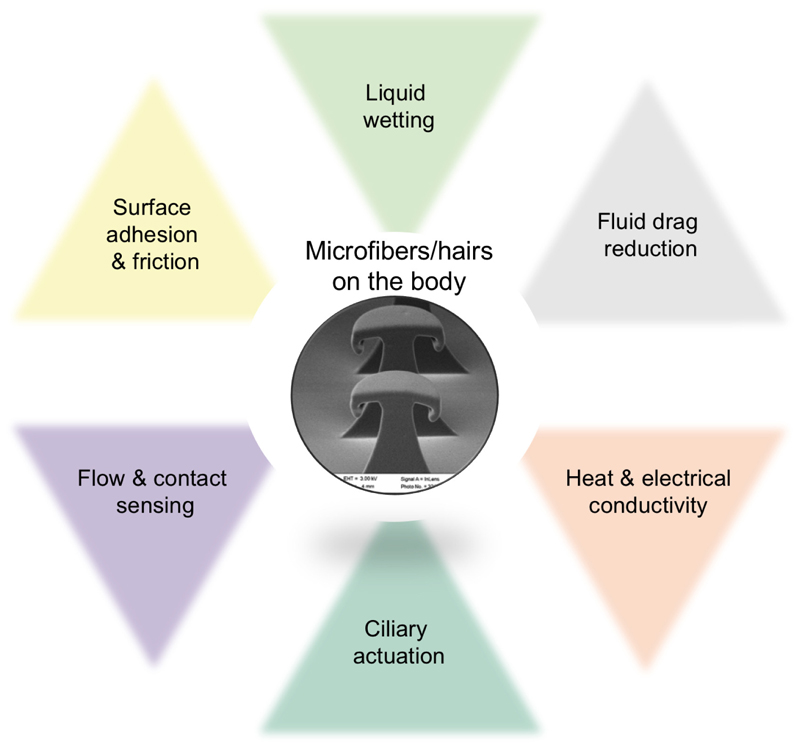
Physical intelligence-based learning and decision-making examples in biological agents. (a) *Stentor roeseli* single-cell organism has a form of sequential decision-making to avoid irritating repeated stimuli (Adapted with permission from [107]. Image credit: Joseph Dexter and Sudhakaran Prabakaran. Copyright 2019, Current Biology). (b) A slime mold (yellow *Physarum polycephalum)* can learn and solve mazes and the traveling salesperson problem in an energy efficient way if a food attractant is placed at target locations (Adapted with permission from [108]. Copyright 2010, Science, AAAS). (c) The hunting cycle of the carnivorous Venus flytraps *(Dionaea muscipula)* (photo credit: Noah Elhart [109]) involves multiple decision-making steps to trap, fully close/seal and digest a landed prey insect.. (For interpretation of the references to color in this figure legend, the reader is referred to the web version of this article.)

Can the agent body make decisions and learn in addition to physical adaptation? Similar to animals with nervous systems, decision-making and learning could also occur in aneural organisms (i.e., organisms with no neurons) [110], such as single- and multi-nucleate cells, where some cells can make decisions rather than just reacting to external stimuli. Therefore, studying and getting inspiration by such biological behavior encoded inside the cell could be a promising decision-making method for PI systems. Cellular decision-making has been widely studied in the systems biology field. As a single-cellular decision-making example, *Stentor roeseli* (a sessile, colorless, trumpet-shaped and ciliated eukaryotic cell) is proved to have a form of sequential decision-making to avoid irritating repeated stimuli [107] (Fig. 4a). In 1906, Herbert S. Jennings [111] described in such cell a hierarchy of diverse behaviors/responses (resting, bending away, ciliary alteration, contraction, and detachment from the holdfast) to repeated stimulation. Moreover, adaptive choice behavior when confronting multiple stimulations [112] could be another inspiration source for encoding decision making physically or biologically in autonomous machines.

Learning is also possible without any neural computation. As an example, a slime mold (yellow *Physarum polycephalum;* an enormous single cell with a large number of nuclei) can learn and solve mazes and the traveling salesperson problem in an energy efficient way if a food attractant is placed at target locations [108, 113] (Fig. 4b). During foraging, the slime mold uses its soft-body branching to crawl on its environment to randomly cover and search for food. Its chemotactic sensing enables to find the food in the target locations. It leaves a trail of translucent extracellular slime to enable an externalized spatial memory, where it retracts its branches that did not find food and leaves behind only the interconnected branches of slime that linked the pieces of food in the shortest path. Thus, they can learn the energy-efficient shortest routes between food sources. Such method could be adapted to physical agents as a PI method by encoding similar taxis, spatial memory, reconfiguration, computation, and information processing behavior in the agent body.

Decision making in touch-sensitive plants, such as carnivorous Venus flytraps *(Dionaea muscipula)* (Fig. 4c), is also an interesting PI method to get inspiration by. Sensory hairs on the surface of the flytrap leaf inner lobes deflect and generate electrical signals (action potential (AP) spikes) on the lobe inner surface when any landed and moving prey insect touches them [114]. Using such evoked AP signals, the plant needs to decide when to partially close the trap fast to capture the prey, when to close and seal the trap fully, and when to start the digestion process. The flytrap decides to close its lobes quickly to capture the prey when the sensory hairs evoke two APs within 30 s. Here, the trap does not close in single AP detection since it needs to guarantee the existence of a landed prey as much as possible, where closing due to a false or a not fully landed prey’s single contact would cause energy and time loss for the plant. Such multiple sensor signal counting is only possible by a chemical memory/clock (cytosolic Ca^2^+ transients/clock induced by each AP) and signaling pathway that triggers the active deformation onset [115]. Ca^2^+ transients due to two AP signals within 30 s trigger a hydraulically driven lobe deformation, which onsets a fast trap closure in about 100 ms, where such fast closure (the fastest motion in the plant kingdom) results from a snap-through buckling instability. Here, the flytrap leaves utilize a PI material structuring method for such unusually fast closure: its each leaf has an asymmetric pre-strain field and a doubly curved slender geometry, inducing a bistable lobe structure with two stable states (open and closed) and a buckling instability [116,117]. As the second decision, using the similar mechanism, if the prey touches three or more times to the sensory hairs while trying to escape from the trap, the jasmonic acid (touch hormone) synthesis is triggered and the trap is fully closed and hermetically sealed. Much larger number of evoked APs due to the prey motion triggers the prey digestion process as the third decision.

### 4.6 Physical intelligence in a large number of physical agents

A single agent is limited in its possible overall intelligence (CI and PI), functionality (e.g., covering a given large task space and manipulating much larger size or number of objects in its operation environment) and complexity. However, multiple (team) or a large number (swarm) of homogeneous or heterogeneous agents can collectively behave to enhance their intelligence, functionality, robustness, fault tolerance and complex behaviors beyond the limits of the individual agents. Furthermore, the coordination of swarm agents could allow much more flexibility than single agents by reconfiguring their formations, behaviors and functions for diverse tasks in highly dynamic changing environments. In addition to CI methods (collective behavior emerging from simple local interaction rules), PI-based coupled local physical interactions between many agents and interactions of agents with the environment could induce collective and complex behaviors, such as self-assembly, self-organization and emergent behavior.

For the agents operating at small length scales, such as milli- and microscale, scaling laws make the surface area- and length-related, short- or long-range physical forces (e.g., fluidic drag, surface tension, adhesion, friction, van der Waals forces) become more dominant than the volume-related physical forces (e.g., inertial forces, weight, buoyancy) [18]. Moreover, the agent motion becomes much more stochastic going down to the cellular size scales due to the Brownian motion and other stochastic effects. Therefore, agents at the small length scales could have long-range fluidic, surface tension and magnetic interactions, which can couple a large number of neighboring agents physically and induce self-organization. For example, a dense motile bacteria swarm can induce self-organized fluidic flow patterns to mix the fluidic media to bring new food source from outside of the swarm to inside so that the bacteria in the swarm center do not perish due to the constantly consumed food [118,119]. Confining surfaces in the environment can play a crucial role in the self-organized patterns [120,121]. Similarly, magnetic microrobot swarms can induce programmable self-assembled or self-organized patterns using the static or dynamic equilibrium of the robot-robot and robot-environment local physical interaction forces to have different collective functions, such as navigating in environments with varying size and shape constraints, manipulating various large-size and complex-shape objects in the environment, and providing a large enough volume and coverage of drugs to a target location inside the human body [122–127].

At the cellular length scales, collective behavior can also be achieved by the physical coupling and coordination of randomly moving tiny agent swarms [128]. For example, living cells aggregate and migrate collectively during the healing of wounds and when cancer spreads. Such biological mechanisms can inspire collective robotic systems, in which deterministic locomotion can be a result of the stochastic movement of many loosely physically coupled agents. Here, stochasticity (randomness) can offer a promising approach to developing large-scale, collective robotic systems that exhibit robust deterministic collective behavior. Such robotic collective can even have a light-driven phototactic motion control [129]. Furthermore, randomly self-propelled particles enclosed in giant unilamellar vesicles can collectively generate various non-equilibrium membrane shapes and active membrane fluctuations [130].

At the insect and centimeter length scales, collective construction and manipulation capabilities and other collective behaviors of termite, honey bee and ant swarms using various PI smart material- and mechanism-based and CI algorithm-based methods can inspire collective robotic construction and other systems [131,132]. In many of these collective systems, a decentralized and stochastic construction approach is used where building cues are extracted from the task environment and provide locally observable positive and negative feedback to the structure growth. Stigmergic coordination (agents affecting the behavior of other agents through environmental modifications or secretions) in such social insect collectives is a common PI method, which can be utilized by robotic agents also.

## 5 Concluding remarks

In addition to CI, it is essential to advance the PI to create autonomous machines to operate in complex real-world environments. PI needs to be specialized for given desired behaviors, environment and functions while CI can be more general purpose. Therefore, evolution has figured out specialized neural cell networks and NI around 500 million years ago, to enable more general-purpose and advanced overall intelligence in animals. In CI-dominated agents, enhancing the PI capability of the agent body would minimize the load of, simplify or complement CI. On the other hand, many biological (e.g., plants, aneural organisms, single cells, soft-bodied organisms, cell collectives) and human-made physical agents (e.g., cell- and insect-scale robots, soft robots, robot collectives) can have dominantly PI to operate in real-world environments. Moreover, in special harsh environments, where extreme conditions can hinder the operation of electronic devices, and cell-scale robots, where there are no on-board CI capabilities, PI is the only option. Bioinspired and abstract methods can create advanced PI capabilities. Although there has been some progress in different aspects of PI methods so far, they are still at a very preliminary, partial and simplistic level and there are still many open issues and topics, including PI-based memory, logic, computation, control, learning, decision-making, and emergent and other collective behaviors. Therefore, PI research is still in its early infancy, and further progress entails highly interdisciplinary collaborations among engineers, physicists, biologists, material scientists, chemists, and computer scientists.

## References

[1] Floreano D, Mattiussi C (2008). Bio-Inspired Artificial Intelligence: Theories, Methods, and Technologies.

[2] Engelbrecht AP (2007). Computational Intelligence: An Introduction.

[3] Hochner B (2008). Octopuses. Curr Biol.

[4] Pfeifer R, Bongard J (2006). How the Body Shapes the Way We Think: A New View of Intelligence.

[5] Barrett L (2011). Beyond the Brain: How Body and Environment Shape Animal and Human Minds.

[6] McEvoy MA, Correll N (2015). Materials that couple sensing, actuation, computation, and communication. Science.

[7] Bhattacharya K, James RD (2005). The material is the machine. Science.

[8] Pfeifer R, Lungarella M, Iida F (2007). Self-organization, embodiment, and biologically inspired robotics. science.

[9] Howard D, Eiben AE, Kennedy DF, Mouret JB, Valencia P, Winkler D (2019). Evolving embodied intelligence from materials to machines. Nat Mach Intell.

[10] Miriyev A, Kovač M (2020). Skills for physical artificial intelligence. Nat Mach Intell.

[11] Raichle ME (2006). The brain's dark energy. Science.

[12] http://www.strandbeest.com.

[13] Routledge Robert, Discoveries & Inventions of the Nineteenth Century.

[14] Preston DJ, Rothemund P, Jiang HJ, Nemitz MP, Rawson J, Suo Z, Whitesides GM (2019). Digital logic for soft devices. Proc Natl Acad Sci.

[15] Duhamel PEJ, Perez-Arancibia CO, Barrows GL, Wood RJ (2012). Biologically inspired optical-flow sensing for altitude control of flapping-wing microrobots. IEEE/ASME Trans Mechatronics.

[16] Sareh P, Chermprayong P, Emmanuelli M, Nadeem H, Kovac M (2018). Ro- torigami: A rotary origami protective system for robotic rotorcraft. Sci Robot.

[17] Mintchev S, Floreano D (2016). Adaptive morphology: A design principle for multimodal and multifunctional robots. IEEE Robot Autom Mag.

[18] Sitti M (2017). Mobile Microrobotics.

[19] Kim S, Clark JE, Cutkosky MR (2006). Isprawl: Design and tuning for highspeed autonomous open-loop running. Int J Robot Res.

[20] Ma KY, Chirarattananon P, Fuller SB, Wood RJ (2013). Controlled flight of a biologically inspired, insect-scale robot. Science.

[21] Murphy MP, Kute C, Mengüç Y, Sitti M (2011). Waalbot II: Adhesion recovery and improved performance of a climbing robot using fibrillar adhesives. Int J Robot Res.

[22] Kim S, Spenko M, Trujillo S, Heyneman B, Santos D, Cutkosky MR (2008). Smooth vertical surface climbing with directional adhesion. IEEE Trans Robot.

[23] Liimatainen V, Drotlef DM, Son D, Sitti M (2020). Liquid-superrepellent bioinspired fibrillar adhesives. Adv Mater.

[24] Kim S, Cheung E, Sitti M (2009). Wet self-cleaning of biologically inspired elastomer mushroom shaped microfibrillar adhesives. Langmuir.

[25] Evans HB, Hamed AM, Gorumlu S, Doosttalab A, Aksak B, Chamorro LP, Castillo L (2018). Engineered bio-inspired coating for passive flow control. Proc Natl Acad Sci.

[26] Tao J, Yu XB (2012). Hair flow sensors: from bio-inspiration to bio-mimicking— a review. Smart Mater Struct.

[27] Dong X, Lum GZ, Hu W, Zhang R, Ren Z, Onck PR, Sitti M (2020). Bioinspired cilia arrays with programmable nonreciprocal motion and metachronal coordination. Sci Adv.

[28] Van Oosten CL, Bastiaansen CW, Broer DJ (2009). Printed artificial cilia from liquid-crystal network actuators modularly driven by light. Nature Mater.

[29] Vilfan M, Potočnik A, Kavčič B, Osterman N, Poberaj I, Vilfan A, Babic D (2010). Self-assembled artificial cilia. Proc Natl Acad Sci.

[30] Lussier Desbiens A, Asbeck AT, Cutkosky MR (2011). Landing, perching and taking off from vertical surfaces. Int J Robot Res.

[31] Cabanach P, Pena-Francesch A, Sheehan D, Bozuyuk U, Yasa O, Borros S, Sitti M (2020). Zwitterionic 3D-printed non-immunogenic stealth microrobots. Adv Mater.

[32] Alapan Y, Yasa O, Schauer O, Giltinan J, Tabak AF, Sourjik V, Sitti M (2018). Soft erythrocyte-based bacterial microswimmers for cargo delivery. Sci Robot.

[33] Rossiter J, Winfield J, Ieropoulos I (2016). Here today, gone tomorrow: biodegradable soft robots. Electroactive Polymer Actuators and Devices (EAPAD) 2016.

[34] Ceylan H, Yasa IC, Yasa O, Tabak AF, Giltinan J, Sitti M (2019). 3D-printed biodegradable microswimmer for theranostic cargo delivery and release. ACS Nano.

[35] Miyashita S, Guitron S, Yoshida K, Li S, Damian DD, Rus D (2016). Ingestible, controllable, and degradable origami robot for patching stomach wounds.

[36] Kang B, Lee Y, Piao T, Ding Z, Wang WD (2021). Robotic soft swim bladder using liquid-vapor phase transition. Mater Horiz.

[37] Shan S, Kang SH, Raney JR, Wang P, Fang L, Candido F, Lewis JA, Bertoldi K (2015). Multistable architected materials for trapping elastic strain energy. Adv Mater.

[38] Felton S, Tolley M, Demaine E, Rus D, Wood R (2014). A method for building self-folding machines. Science.

[39] Miyashita S, Guitron S, Li S, Rus D (2017). Robotic metamorphosis by origami exoskeletons. Sci Robot.

[40] Saito K, Perez-de la Fuente R, Arimoto K, Seong ah Y, Aonuma H, Niiyama R, You Z (2020). Earwig fan designing: Biomimetic and evolutionary biology applications. Proc Natl Acad Sci.

[41] Göttler C, Elflein K, Siegwart R, Sitti M (2021). Spider origami: Folding principle of jumping spider leg joints for bioinspired fluidic actuators. Adv Sci.

[42] Rafsanjani A, Zhang Y, Liu B, Rubinstein SM, Bertoldi K (2018). Kirigami skins make a simple soft actuator crawl. Sci Robot.

[43] Cheng YC, Lu HC, Lee X, Zeng H, Priimagi A (2020). Kirigami-based light- induced shape-morphing and locomotion. Adv Mater.

[44] Paul C, Valero-Cuevas FJ, Lipson H (2006). Design and control of tensegrity robots for locomotion. IEEE Trans Robot.

[45] Wen L, Pan F, Ding X (2020). Tensegrity metamaterials for soft robotics. Sci Robot.

[46] Bartlett NW, Tolley MT, Overvelde JT, Weaver JC, Mosadegh B, Bertoldi K, Whitesides GM, Wood RJ (2015). A 3D-printed, functionally graded soft robot powered by combustion. Science.

[47] Wehmann H-N, Heepe L, Gorb SN, Engels T, Lehmann F-O (2019). Local deformation and stiffness distribution in fly wings. Biol Open.

[48] Hansen KM, Thundat T (2005). Microcantilever biosensors. Methods.

[49] Morin SA, Shepherd RF, Kwok SW, Stokes AA, Nemiroski A, Whitesides GM (2012). Camouflage and display for soft machines. Science.

[50] Hu W, Lum GZ, Mastrangeli M, Sitti M (2018). Small-scale soft-bodied robot with multimodal locomotion. Nature.

[51] Kim Y, Yuk H, Zhao R, Chester SA, Zhao X (2018). Printing ferromagnetic domains for untethered fast-transforming soft materials. Nature.

[52] Ren Z, Hu W, Dong X, Sitti M (2019). Multi-functional soft-bodied jellyfish-like swimming. Nature Commun.

[53] Wang W, Yao L, Cheng CY, Zhang T, Atsumi H, Wang L, Wang G, Anilionyte O, Steiner H, Ou J, Zhou K (2017). Harnessing the hygroscopic and biofluorescent behaviors of genetically tractable microbial cells to design biohybrid wearables. Sci Adv.

[54] Lee YW, Ceylan H, Yasa IC, Kilic U, Sitti M (2021). 3D-printed multi-stimuli-responsive mobile micromachines. ACS Appl Mater Interfaces.

[55] McCoy CP, Brady C, Cowley JF, McGlinchey SM, McGoldrick N, Kinnear D-J, Andrews GP, Jones DS (2010). Triggered drug delivery from biomaterials. Expert Opin Drug Deliv.

[56] Sirsi SR, Borden MA (2014). State-of-the-art materials for ultrasound-triggered drug delivery. Adv Drug Deliv Rev.

[57] Bozuyuk U, Yasa O, Yasa IC, Ceylan H, Kizilel S, Sitti M (2018). Light-triggered drug release from 3D-printed magnetic chitosan microswimmers. ACS Nano.

[58] Churchill CB, Shahan DW, Smith SP, Keefe AC, McKnight GP (2016). Dynamically variable negative stiffness structures. Sci Adv.

[59] Semini C, Barasuol V, Boaventura T, Frigerio M, Focchi M, Caldwell DG, Buchli J (2015). Towards versatile legged robots through active impedance control. Int J Robot Res.

[60] Rich S, Jang SH, Park YL, Majidi C (2017). Liquid metal-conductive thermoplastic elastomer integration for low-voltage stiffness tuning. Adv Mater Technol.

[61] Hines L, Arabagi V, Sitti M (2012). Shape memory polymer-based flexure stiffness control in a miniature flapping-wing robot. IEEE Trans Robot.

[62] Wang L, Yang Y, Chen Y, Majidi C, Iida F, Askounis E, Pei Q (2018). Controllable and reversible tuning of material rigidity for robot applications. Mater Today.

[63] Maggi C, Saglimbeni F, Dipalo M, De Angelis F, Di Leonardo R (2015). Micromotors with asymmetric shape that efficiently convert light into work by thermocapillary effects. Nature Commun.

[64] Amador GJ, Ren Z, Tabak AF, Alapan Y, Yasa O, Sitti M (2019). Temperature gradients drive bulk flow within microchannel lined by fluid-fluid interfaces. Small.

[65] Pena-Francesch A, Giltinan J, Sitti M (2019). Multifunctional and biodegradable self-propelled protein motors. Nature Commun.

[66] Crowdy D (2020). Collective viscous propulsion of a two-dimensional flotilla of marangoni boats. Phys Rev Fluids.

[67] Li W, Wu X, Qin H, Zhao Z, Liu H (2016). Light-driven and light-guided microswimmers. Adv Funct Mater.

[68] Sridhar V, Podjaski F, Kröger J, Jiménez-Solano A, Park BW, Lotsch BV, Sitti M (2020). Carbon nitride-based light-driven microswimmers with intrinsic photocharging ability. Proc Natl Sci Acad.

[69] Han M, Wang H, Yang Y, Liang C, Bai W, Yan Z, Li H, Xue Y, Wang X, Akar B, Zhao H (2019). Three-dimensional piezoelectric polymer microsystems for vibrational energy harvesting, robotic interfaces and biomedical implants. Nat Electron.

[70] Pu X, Liu M, Chen X, Sun J, Du C, Zhang Y, Zhai J, Hu W, Wang ZL (2017). Ultrastretchable, transparent triboelectric nanogenerator as electronic skin for biomechanical energy harvesting and tactile sensing. Sci Adv.

[71] Shi H, Liu Z, Mei X (2020). Overview of human walking induced energy harvesting technologies and its possibility for walking robotics. Energies.

[72] Mostafalu P, Sonkusale S (2014). Flexible and transparent gastric battery: Energy harvesting from gastric acid for endoscopy application. Biosens Bioelectron.

[73] Lee KB (2005). Urine-activated paper batteries for biosystems. J Micromech Microeng.

[74] Xiao X, Xia HQ, Wu R, Bai L, Yan L, Magner E, Cosnier S, Lojou E, Zhu Z, Liu A (2019). Tackling the challenges of enzymatic (bio) fuel cells. Chem Rev.

[75] Hager MD (2017). Self-healing materials. Handbook of Solid State Chemistry.

[76] White SR, Sottos NR, Geubelle PH, Moore JS, Kessler MR, Sriram SR, Brown EN, Viswanathan S (2001). Autonomic healing of polymer composites. Nature.

[77] Pena-Francesch A, Jung H, Demirel MC, Sitti M (2020). Biosynthetic self-healing materials for soft machines. Nature Mater.

[78] Terryn S, Brancart J, Lefeber D, Van Assche G, Vanderborght B (2017). Self-healing soft pneumatic robots. Sci Robot.

[79] Giachini PAGS, Gupta SS, Wang W, Wood D, Yunusa M, Baharlou E, Sitti M, Menges A (2020). Additive manufacturing of cellulose-based materials with continuous, multidirectional stiffness gradients. Sci Adv.

[80] Liu D, Tarakanova A, Hsu CC, Yu M, Zheng S, Yu L, Liu J, He Y, Dunstan DJ, Buehler MJ (2019). Spider dragline silk as torsional actuator driven by humidity. Sci Adv.

[81] Kuenstler AS, Chen Y, Bui P, Kim H, DeSimone A, Jin L, Hayward RC (2020). Blueprinting photothermal shape-morphing of liquid crystal elastomers. Adv Mater.

[82] Davidson ZS, Shahsavan H, Aghakhani A, Guo Y, Hines L, Xia Y, Yang S, Sitti M (2019). Monolithic shape-programmable dielectric liquid crystal elastomer actuators. Sci Adv.

[83] Guo Y, Shahsavan H, Sitti M (2020). 3D microstructures of liquid crystal networks with programmed voxelated director fields. Adv Mater.

[84] Ware TH, McConney ME, Wie JJ, Tondiglia VP, White TJ (2015). Voxelated liquid crystal elastomers. Science.

[85] Burr GW (2019). A role for analogue memory in AI hardware. Nat Mach Intell.

[86] Bagheri M, Poot M, Li M, Pernice WP, Tang HX (2011). Dynamic manipulation of nanomechanical resonators in the high-amplitude regime and non-volatile mechanical memory operation. Nature Nanotechnol.

[87] Chen T, Pauly M, Reis PM (2021). A reprogrammable mechanical metamaterial with stable memory. Nature.

[88] Yasuda H, Tachi T, Lee M, Yang J (2017). Origami-based tunable truss structures for non-volatile mechanical memory operation. Nature Commun.

[89] Vladimirov N, Sourjik V (2009). Chemotaxis: how bacteria use memory. Biol Chem.

[90] Wehner M, Truby RL, Fitzgerald DJ, Mosadegh B, Whitesides GM, Lewis JA, Wood RJ (2016). An integrated design and fabrication strategy for entirely soft, autonomous robots. Nature.

[91] Prakash M, Gershenfeld N (2007). Microfluidic bubble logic. Science.

[92] Katsikis G, Cybulski JS, Prakash M (2015). Synchronous universal droplet logic and control. Nat Phys.

[93] Woods D, Doty D, Myhrvold C, Hui J, Zhou F, Yin P, Winfree E (2019). Diverse and robust molecular algorithms using reprogrammable DNA self-assembly. Nature.

[94] Park W, Zhuang J, Yasa O, Sitti M (2017). Multifunctional bacteria-driven microswimmers for targeted active drug delivery. ACS Nano.

[95] Alapan Y, Yasa O, Yigit B, Yasa IC, Erkoc P, Sitti M (2019). Microrobotics and microorganisms: Biohybrid autonomous cellular robots. Annu Rev Control Robot Auton Syst.

[96] Felfoul O, Mohammadi M, Taherkhani S, De Lanauze D, Xu YZ, Loghin D, Essa S, Jancik S, Houle D, Lafleur M, Gaboury L (2016). Magneto-aerotactic bacteria deliver drug-containing nanoliposomes to tumour hypoxic regions. Nature Nanotechnol.

[97] Yasa O, Erkoc P, Alapan Y, Sitti M (2018). Microalga-powered microswimmers toward active cargo delivery. Adv Mater.

[98] Grinthal A, Aizenberg J (2013). Adaptive all the way down: building responsive materials from hierarchies of chemomechanical feedback. Chem Soc Rev.

[99] Jiang Y, Korpas LM, Raney JR (2019). Bifurcation-based embodied logic and autonomous actuation. Nature Commun.

[100] Coyle S, Majidi C, LeDuc P, Hsia KJ (2018). Bio-inspired soft robotics: Material selection, actuation, and design. Extreme Mech Lett.

[101] Asbeck AT, Kim S, Cutkosky MR, Provancher WR, Lanzetta M (2006). Scaling hard vertical surfaces with compliant microspine arrays. Int J Robot Res.

[102] Abad SA, Herzig N, Sadati SMH, Nanayakkara T (2019). Significance of the compliance of the joints on the dynamic slip resistance of a bioinspired hoof. IEEE Trans Robot.

[103] Libby T, Moore TY, Chang-Siu E, Li D, Cohen DJ, Jusufi A, Full RJ (2012). Tail-assisted pitch control in lizards, robots and dinosaurs. Nature.

[104] Saab W, Rone WS, Ben-Tzvi P (2018). Robotic tails: a state-of-the-art review. Robotica.

[105] Brown E, Rodenberg N, Amend J, Mozeika A, Steltz E, Zakin MR, Lipson H, Jaeger HM (2010). Universal robotic gripper based on the jamming of granular material. Proc Natl Acad Sci.

[106] Song S, Sitti M (2014). Soft grippers using micro-fibrillar adhesives for transfer printing. Adv Mater.

[107] Dexter JP, Prabakaran S, Gunawardena J (2019). A complex hierarchy of avoidance behaviors in a single-cell eukaryote. Curr Biol.

[108] Tero A, Takagi S, Saigusa T, Ito K, Bebber DP, Fricker MD, Yumiki K, Kobayashi R, Nakagaki T (2010). Rules for biologically inspired adaptive network design. Science.

[109] https://commons.wikimedia.org/wiki/File:Venus_Flytrap_showing_trigger_hairs.jpg.

[110] Eisenstein EM (1975). Aneural Organisms in Neurobiology.

[111] Sonneborn TM (1975). Herbert Spencer Jennings: 1868-1947. Biographical Memoirs.

[112] Reid CR, Garnier S, Beekman M, Latty T (2015). Information integration and multiattribute decision making in non-neuronal organisms. Anim Behav.

[113] Reid R, Latty T, Dussutour A, Beekman M (2012). Slime mold uses an externalized spatial memory to navigate in complex environments. Proc Natl Acad Sci.

[114] Scherzer S, Federle W, Al-Rasheid KAS, Hedrich R (2019). Venus flytrap trigger hairs are micronewton mechano-sensors that can detect small insect prey. Nature Plants.

[115] Hedrich R, Neher E (2018). Venus flytrap: how an excitable, carnivorous plant works. Trends Plant Sci.

[116] Forterre Y, Skotheim JM, Dumais J, Mahadevan L (2005). How the venus flytrap snaps. Nature.

[117] Epstein E, Yoon J, Madhukar A, Hsia KJ, Braun PV (2015). Colloidal particles that rapidly change shape via elastic instabilities. Small.

[118] Kearns B (2010). A field guide to bacterial swarming motility. Nat Rev Microbiol.

[119] Wioland H, Woodhouse FG, Dunkel J, Goldstein RE (2016). Ferromagnetic and antiferromagnetic order in bacterial vortex lattices. Nat Phys.

[120] Wioland H, Woodhouse FG, Dunkel J, Kessler JO, Goldstein RE (2013). Confinement stabilizes a bacterial suspension into a spiral vortex. Phys Rev Lett.

[121] Culha U, Davidson ZS, Mastrangeli M, Sitti M (2020). Statistical reprogramming of macroscopic self-assembly with dynamic boundaries. Proc Natl Acad Sci.

[122] Dong X, Sitti M (2020). Controlling two-dimensional collective formation and cooperative behavior of magnetic microrobot swarms. Int J Robot Res.

[123] Palagi S, Fischer P (2018). Bioinspired microrobots. Nat Rev Mater.

[124] Xie H, Sun M, Fan X, Lin Z, Chen W, Wang L, Dong L, He Q (2019). Reconfigurable magnetic microrobot swarm: Multimode transformation, locomotion, and manipulation. Sci Robot.

[125] Yu J, Jin D, Chan KF, Wang Q, Yuan K, Zhang L (2019). Active generation and magnetic actuation of microrobotic swarms in bio-fluids. Nature Commun.

[126] Wang W, Giltinan J, Zakharchenko S, Sitti M (2017). Dynamic and programmable self-assembly of micro-rafts at the air-water interface. Sci Adv.

[127] Yigit B, Alapan Y, Sitti M (2020). Cohesive self-organization of mobile microrobotic swarms. Soft Matter.

[128] Sitti M (2019). Robotic collectives inspired by biological cells. Nature.

[129] Li S, Batra R, Brown D, Chang HD, Ranganathan N, Hoberman C, Rus D, Lipson H (2019). Particle robotics based on statistical mechanics of loosely coupled components. Nature.

[130] Vutukuri HR, Hoore M, Abaurrea-Velasco C, van Buren L, Dutto A, Auth T, Fedosov DA, Gompper G, Vermant J (2020). Active particles induce large shape deformations in giant lipid vesicles. Nature.

[131] Petersen KH, Napp N, Stuart-Smith R, Rus D, Kovac M (2019). A review of collective robotic construction. Sci Robot.

[132] Dorigo M, Theraulaz G, Trianni V (2020). Reflections on the future of swarm robotics. Sci Robot.

